# Lesion detection and assessment of extrahepatic findings in abdominal MRI using hepatocyte specific contrast agents – comparison of Gd-EOB-DTPA and Gd-BOPTA

**DOI:** 10.1186/1471-2342-13-10

**Published:** 2013-03-18

**Authors:** Kristina I Ringe, Daniel T Boll, Daniela B Husarik, Mustafa R Bashir, Rajan T Gupta, Elmar M Merkle

**Affiliations:** 1 Department of Diagnostic and Interventional Radiology, Hannover Medical School, Carl-Neuberg Str. 1, Hannover, 30625, Germany; 2 Department of Radiology, Duke University Medical Center, Box 3808, Durham, North Carolina, 27710, USA; 3 University Hospital Basel, Radiology and Nuclear Medicine, Petersgraben 4, Basel, CH-4031, Switzerland

**Keywords:** Gd-EOB-DTPA, Gadoxetate disodium, Gd-BOPTA, Extrahepatic

## Abstract

**Background:**

To evaluate the contrast agent performance of Gd-EOB-DTPA and Gd-BOPTA for detection and assessment of extrahepatic findings, semi-quantitatively and qualitatively.

**Methods:**

13 patients with 19 extrahepatic lesions underwent liver MRI with Gd-EOB-DTPA and Gd-BOPTA. Quantitative and relative SNR measurements were performed in each dataset in the arterial and portalvenous phase within the extrahepatic lesion, aorta, inferior vena cava, portal vein, spleen, pancreas and renal cortex. Further, relative CNR measurements were performed. Three readers assessed contrast quality using a five-point scale and choosing the preferred image dataset. Statistical analysis consisted of a Student’s t-test with *p* < 0.05 deemed significant, a weighted kappa statistic for assessment of interobserver variability and an ROC analysis.

**Results:**

Mean SNR after injection of Gd-BOPTA was significantly higher compared with Gd-EOB-DTPA for all measurements (*p* < 0.05). Mean relative SNR was also higher for Gd-BOPTA, but without being statistically significant. There was no significant difference in relative CNR. Interobserver agreement for selection of image preference was moderate (mean weighted kappa 0.485). The area under the curve for the ROC-analysis regarding contrast agent performance was 0.464.

**Conclusion:**

Even though mean SNR is significantly higher after injection of Gd-BOPTA compared with Gd-EOB-DTPA, there is no significant difference in relative CNR with extrahepatic lesions being assessed equally well. Visual impression may differ after injection of Gd-EOB-DTPA, but does not influence image interpretation. Extrahepatic findings can be assessed similarly to MRI after injection of Gd-BOPTA.

## Background

Gadoxetate disodium (Gd-EOB-DTPA, gadoxetic acid, Eovist® or Primovist®, Bayer HealthCare, Wayne, NJ) is a liver-specific contrast agent, which has recently become available for detection and characterization of focal hepatic lesions. Pharmacokinetic and pharmacodynamic properties of Gd-EOB-DTPA differ not only from other non-specific contrast agents used in MR imaging, but also from more established liver-specific contrast agents such as Gd-BOPTA (gadobenate dimeglumine, Multihance®, Bracco, Princeton, NJ) [1,2].

In contrast to pure extracellular contrast agents, both substances show a partial specific uptake by hepatocytes and subsequently biliary excretion, allowing for data acquisition in the hepatocyte phase in addition to the usual dynamic phases. Whereas hepatic uptake and biliary elimination for gadobenate dimeglumine is only 3-5%, excretion through the hepatobiliary pathway is approximately 50% for gadoxetate disodium [1,3]. In addition, the characteristic pharmacodynamic properties of Gd-EOB-DTPA allow a lower dosage compared with other gadolinium chelates [4]. A dosage of 0.025 mmol/kg body weight is approved by the FDA. This dosage is equivalent to one quarter of the gadolinium dose recommended for all other MRI contrast agents approved by the FDA for liver imaging. However, the approved dosage of 0.025 mmol/kg body weight for Gd-EOB-DTPA is currently under debate with some radiologists preferring twice that dosage.

Recapitulating, it is known that the distinct pharmacokinetic and pharmacodynamic properties of Gd-EOB-DTPA may result in a different image appearance especially of primary and secondary liver lesions [5,6], as well as other parenchymal organs and vessels [7,8], compared with other established MRI contrast agents such as Gd-BOPTA [9]. As comprehensive MR imaging of the liver usually includes imaging of the upper abdomen, this leads to the question, whether image interpretation of incidental extrahepatic findings may be influenced unknowingly. It has been suggested that further clinical studies in patients with various tumors are needed to clarify this circumstance and to establish more routine [8]. Thus, the purpose of our study was to evaluate the contrast agent performance of Gd-EOB-DTPA and Gd-BOPTA for detection and assessment of extrahepatic findings both, semi-quantitatively and qualitatively.

## Methods

This retrospective study was approved from the local institutional review board of Duke University Medical Center with a waiver of consent granted.

### Patients

A total of 13 patients (8 male, 5 female; mean age 57.4 years, range 37–73 years) were included in this study, who met the inclusion criteria. The patients were chosen from a database consisting of a total of 552 patients in whom MRI was performed including the administration of Gd-EOB-DTPA at our institution between October 2008 and January 2010. All patients were referred for liver MR imaging, including administration of a hepatocyte specific contrast agent. Due to a change in clinical routine patients received different contrast agents over time and for clinical follow-up. Inclusion criteria were as follows: age of at least 18 years, availability of a comparison study within one year and administration of either Gd-EOB-DTPA or Gd-BOPTA, and presence of an extrahepatic finding seen on both imaging studies.

The extrahepatic lesions were as follows: 4 adrenal lesions (adenoma n = 3, metastasis n = 1), 5 kidney lesions (angiomyolipoma n = 1, metastasis n = 4), 5 splenic lesions (hemangioma n = 5), 2 pulmonary lesions (hamartoma n = 2), 1 pancreatic lesion (microcystic cystadenoma n = 1), 2 spinal lesions (metastasis n = 2). Parenchymal lesions showed no change in size (mean lesion size: 2.15 cm; range: 1–4.32 cm) and appearance between comparative studies. Patient characteristics are summarized in Table 1.

**Table 1:** Patient characteristics and assessed extrahepatic findings

**Gender (male / female)**	**8 / 5**
Age (years)	37-73 (mean 57.4)
Extrahepatic lesion	
Adrenal adenoma	3
Adrenal metastasis	1
Angiomyolipoma kidney	1
Kidney metastasis	4
Splenic hemangioma	5
Pulmonary hamartoma	2
Pancreatic cystadenoma	1
Spinal metastasis	2

### MR imaging technique

MR examinations were all performed on 1.5 T systems. Including the administration of Gd-EOB-DTPA examinations were performed on following scanners: Magnetom Avanto (Siemens, Erlangen, Germany), n = 5; Signa HDx (GE Healthcare, Milwaukee, USA), n = 8. Comparison studies including the administration of Gd-BOPTA were performed on the same scanners (Magnetom Avanto; Siemens (n = 5) or Signa HDx; GE Healthcare (n = 8)).

In all patients, dedicated multidetector surface coils that covered the abdomen were used. All patients underwent a clinical routine image protocol of the liver including the administration of either 0.025 mmol/ kg Gd-EOB-DTPA or 0.1 mL/ kg Gd-BOPTA at a rate of 2 mL/ sec followed by a saline flush using a dual power injector. The MR pulse sequence protocol for all examinations included a T2w HASTE (half-Fourier single shot turbo spin-echo) and a T1w gradient dual echo sequence before contrast injection, as well as a dynamic contrast series including the acquisition of a triple arterial phase (3D T1w gradient echo sequence) with a fixed scan delay (15 seconds in patients <60 years and 20 seconds in patients >60 years, respectively) and a portal venous phase (3D T1w gradient echo sequence) after contrast injection, which was acquired approximately 15–20 seconds after completion after the arterial phase data acquisition. These imaging sequences and parameters were the same between imaging platforms and comparative studies.

### Image evaluation

a. Quantitative analysis: In each patient signal-to-noise ratio (SNR; signal intensity / standard deviation noise) measurements were obtained in each dataset (Gd-EOB-DTPA and Gd-BOPTA) in the arterial and portal venous phase in corresponding positions. Noise estimates were derived in each dataset from a region outside the body in the vicinity of the liver. Measurements were all conducted by two radiologists in consensus (KIR, fifth year radiology resident and EMM, fellowship-trained radiologist with a focus on abdominal imaging and 13 years of experience after board certification) who where blinded to the type of dataset. By placing an oval region of interest (ROI) measurements were performed in the aorta, inferior vena cava, portal vein, pancreas, spleen, renal cortex and the extrahepatic lesion. The size of the ROI was adjusted to the size of the lesion and the same for comparative studies. In addition, relative SNR ((SNR _post contrast_ - SNR _pre contrast_) / SNR _pre contrast_) and relative contrast to noise (CNR; relative SNR _lesion_ - relative SNR _organ_) measurements were obtained in the same position of each dataset and examination.

b. Qualitative analysis: Of each dataset (Gd-EOB-DTPA and Gd-BOPTA) representative T1w non contrast, arterial and portal venous phase images were prepared for blinded reading and opposed for comparison (dataset A and dataset B). Three independent readers performed the qualitative image analysis in terms of assessing lesion detectability and contrast quality using a five-point scale: 1, non diagnostic; 2, poor; 3, acceptable; 4, good; 5, very good. The group of readers comprised a junior abdominal imaging attending one year post fellowship training (RTG), an abdominal imaging fellow (MRB) and a fifth year radiology resident (DBH). In addition, each reader was asked to choose the preferred imaging dataset.

c. Statistical analysis: Statistical analysis was performed with SPSS software (version 17; SPSS, Chicago, Illinois). Results of the quantitative analyses were compared with a two-sided unpaired student’s T-test, with *p* < 0.05 deemed significant. Interobserver variability was assessed by means of a weighted kappa statistic and an ROC analysis. Interobserver agreement was classified as follows: a κ value of less than 0.20 indicated poor agreement; a κ value of 0.21-0.40, fair agreement; a κ value of 0.41-0.60, moderate agreement; a κ value of 0.61-0.80, good agreement; a κ value of 0.81-1.00, excellent agreement [10].

## Results

**Figure 1:**
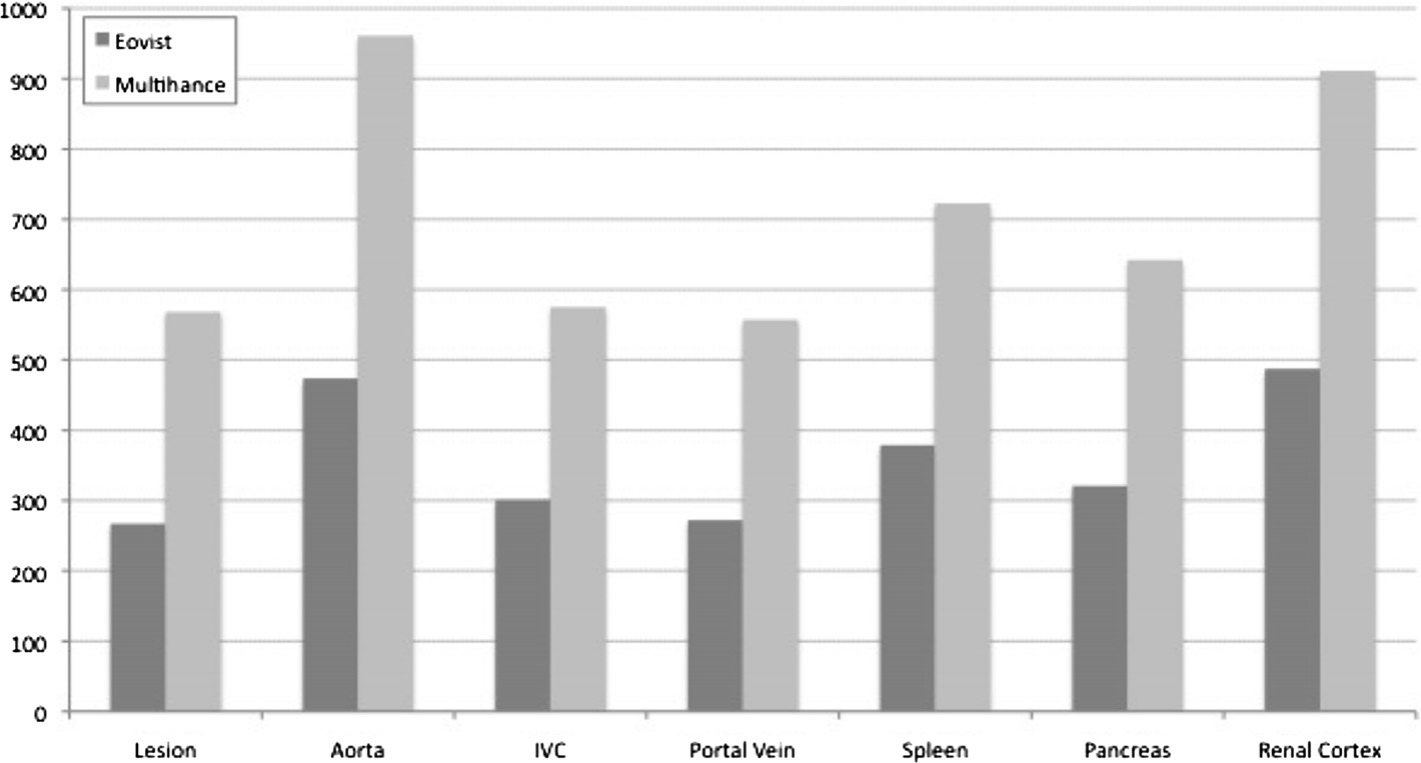
**Comparison of SNR measurements using Gd-EOB-DTPA (Eovist**^**→**^**, Bayer HealthCare, Wayne, NJ) and Gd-BOPTA (Multihance**^**→**^**, Bracco, Princeton, NJ) in the arterial phase.** Shown is the mean SNR for the extrahepatic lesion, as well as for different vessels and organs.

**Figure 2:**
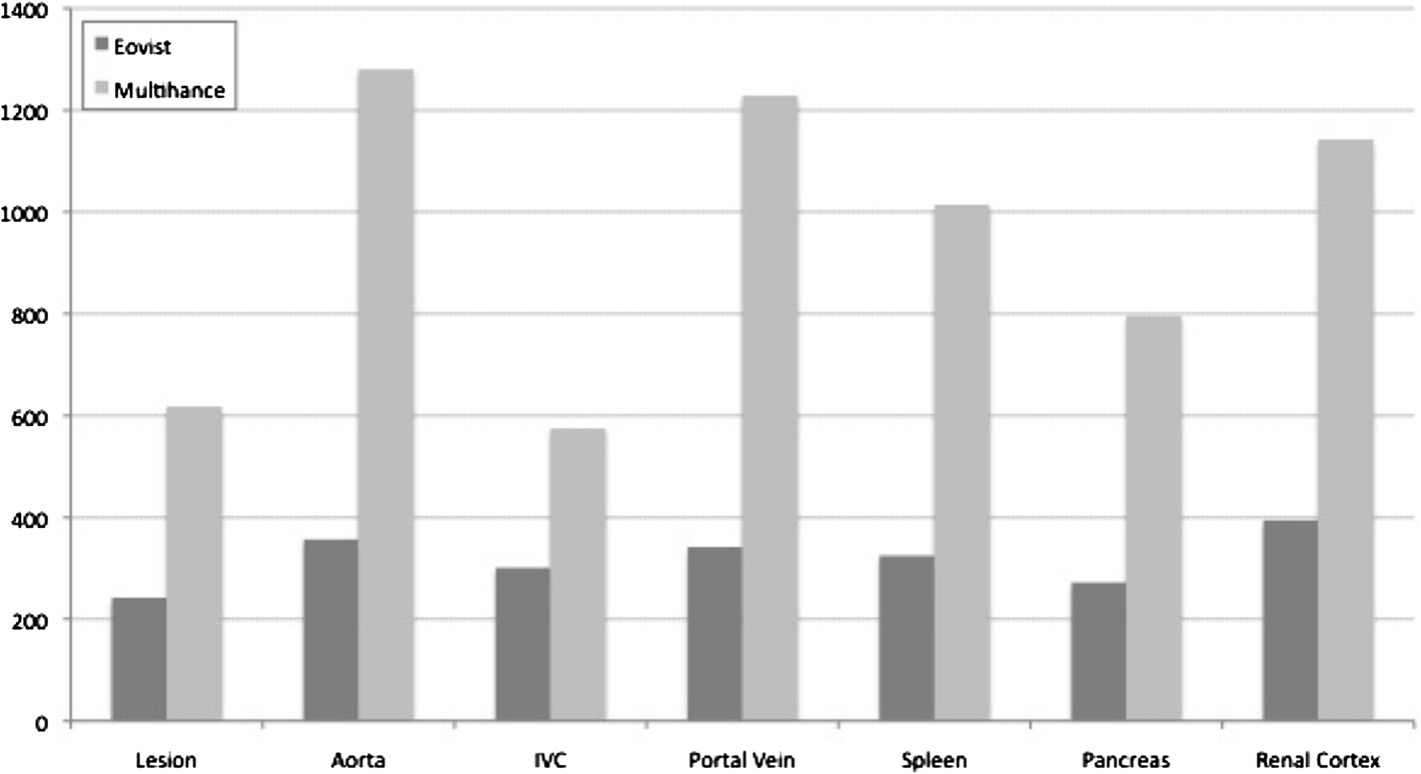
**Comparison of SNR measurements using Gd-EOB-DTPA (Eovist**^**→**^**, Bayer HealthCare, Wayne, NJ) and Gd-BOPTA (Multihance**^**→**^**, Bracco, Princeton, NJ) in the portal venous phase.** Shown is the mean SNR for the extrahepatic lesion, as well as for different vessels and organs.

**Figure 3:**
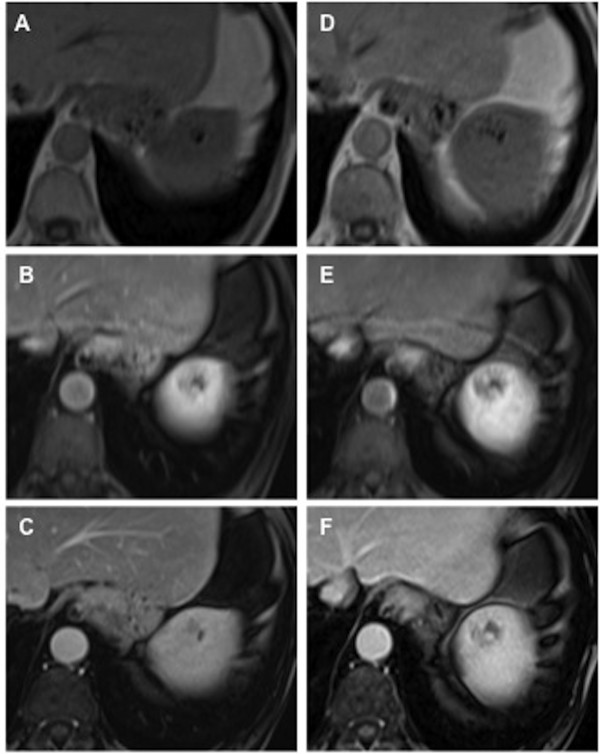
**Comparison of Gd-BOPTA (A-C) and Gd-EOB-DTPA (D-E) for detection and characterization of a splenic hemangioma.** Pre contrast T1w images (**A**,**D**; top row), arterial phase images (**B**,**E**; middle row) and portal venous phase images (**C**,**F**; bottom row).

All patients underwent MR imaging of the upper abdomen using Gd-BOPTA and Gd-EOB-DTPA. Mean time interval between both examinations was 165 days (range 23–265 days).

a. Quantitative analysis: Mean SNR after injection of Gd-BOPTA was significantly higher compared with Gd-EOB-DTPA for all lesions, vessels and organs, in the arterial as well as in the portal venous phase (*p* < 0.05) (Figures 1, 2). Mean SNR for assessed lesions in the arterial phase was 266.7 (range 50–528) for Gd-EOB-DTPA and 567.5 (range 52–1956) for Gd-BOPTA, respectively. Mean SNR for evaluated lesions in the portal venous phase was 221.4 (range 72–428) for Gd-EOB-DTPA and 617.6 (range 88–2502) for Gd-BOPTA, respectively. Mean relative SNR was also higher for Gd-BOPTA, but without being statistically significant (e.g. mean relative SNR for assessed lesions in the arterial phase was 0.9 (range 0.1-3.3) for Gd-EOB-DTPA and 1.0 (range 0.1-3,8) for Gd-BOPTA; mean relative SNR for evaluated lesions in the portal venous phase was 0.6 (range 0.1-2.4) for Gd-EOB-DTPA and 1.1 (range 0.1-4,5) for Gd-BOPTA, respectively). For all measurements there was no significant difference in relative CNR, neither in the arterial nor in the portal venous phase (*p* > 0.05).

b. Qualitative analysis: For all readers there were no significant differences in image quality comparing imaging with Gd-BOPTA and Gd-EOB-DTPA (Figure 3). Interobserver variability regarding the assessment of image quality was fair for Gd-EOB-DTPA and Gd-BOPTA with a mean weighted kappa of 0.245 and 0.316, respectively. Interobserver agreement for selection of image preference was moderate (mean weighted kappa 0.485). The area under the curve for the ROC-analysis was 0.464, implying that there was no significant difference comparing imaging with Gd-EOB-DTPA and Gd-BOPTA.

## Discussion

Extracellular contrast agents, such as gadopentetate dimeglumine (Gd-DTPA, Magnevist, Bayer HealthCare), have been in clinical use for more than two decades and are well established. More recently, liver-specific contrast agents have become available for the detection and characterization of focal hepatic lesions. Whereas Gd-BOPTA has been approved for liver imaging by the European authorities several years ago and certain experience and routine is available, Gd-EOB-DTPA is fairly new and has gained FDA approval only in 2008.

Brismar et al. compared liver vessel and liver parenchymal enhancement after the injection of Gd-EOB-DTPA and Gd-BOPTA using a bolus technique in ten healthy volunteers. Results showed a higher maximum of enhancement of the hepatic artery, portal vein and middle hepatic vein during the arterial, portal venous phase and delayed phase for Gd-BOPTA, while there was no significant difference in liver parenchymal contrast enhancement [7]. So far only few studies have compared the performance of Gd-EOB-DTPA with other more established contrast agents regarding the assessment of extrahepatic tissues and vessels. Kühn et al. compared the enhancement patterns of solid organs and the abdominal aorta after the injection of gadobutrol and gadoxetate disodium in 50 patients. Mean enhancement indexes were higher for gadobutrol except for the abdominal aorta, and it has been suggested that early dynamic MRI of the upper abdomen benefits from the higher gadolinium concentration of gadobutrol than in the organ-specific contrast agent gadoxetic acid [11]. However, patients in the two compared contrast agents groups were not the same. These results were similar to those gained by Zizka et al. earlier [12]. A more recent intraindividual comparison of liver, abdominal and pulmonary vessel enhancement in staging for rectal carcinoma showed comparable contrast enhancement after gadoxetic acid to gadobutrol [13]. Tamada et al. compared enhancement patterns of solid abdominal organs and vessels in 13 healthy volunteers after the injection of Gd-DTPA (Magnevist) and Gd-EOB-DTPA. It has been proposed that lower arterial vascular and parenchymal enhancement with Gd-EOB-DTPA as compared with GD-DTPA may require reassessment of its dose, despite the higher late venous phase liver parenchymal enhancement [8].

To the best of our knowledge, no study has so far compared the enhancement effect of extrahepatic findings in MR imaging with Gd-EOB-DTPA and Gd-BOPTA. Our results show that even though mean SNR in extrahepatic lesions, vessels and organs is significantly higher after the injection of Gd-BOPTA compared with Gd-EOB-DTPA, there is no significant difference in relative CNR with extrahepatic lesions being assessed adequately. Thus, visual impression may differ after injection of Gd-EOB-DTPA, but does not influence image interpretation, and extrahepatic findings can be assessed similarly to MRI after injection of Gd-BOPTA. The circumstance that interobserver variability for Gd-BOPTA is slightly better than for Gd-EOB-DTPA may be justified to some extent by the fact, that gadoxetate disodium is a relatively new contrast agent resulting in a different image impression and that we are in the process of gaining more routine [5-8].

Our study has a number of limitations. The time interval between MRI examination with Gd-EOB-DTPA and Gd-BOPTA was fairly long in some patients (mean 165 days). On the other hand, evaluated parenchymal lesions showed no change in size and appearance between comparative studies, so that evaluation especially of metastatic lesions could not be influenced by these factors. Also, comparative studies were performed on the same scanners using identical imaging parameters. Another limitation is the small sample size and heterogeneity of incidentally detected lesions (malignant as well as benign). As the same lesion was evaluated on two different studies, we think that this intralesional comparison is valid.

## Conclusions

In conclusion, hepatocyte-specific contrast agents are increasingly used for comprehensive MR imaging of the liver. Incidental extrahepatic parenchymal findings are common and need to be appraised when imaged. It is known that the distinct pharmacokinetic and pharmacodynamic properties of Gd-EOB-DTPA may result in a different image appearance especially of primary and secondary liver lesions. Therefore assessment of extrahepatic findings may unknowingly be influenced as well, and a comparison of contrast agent performance is clinically important. The results of our study show, that visual impression of incidental extrahepatic findings may differ after injection of Gd-EOB-DTPA compared with Gd-BOPTA, but does not influence image interpretation. Reliable assessment of extrahepatic findings is feasible using the hepatocyte-specific contrast agent Gd-EOB-DTPA, although results need to be validated in future studies with larger patient cohorts.

## Competing interests

The authors declare that they have no competing interests.

## Authors’ contributions

KIR and EMM conceived and designed the experiments. KIR, DTB, DBH, MRB, RTG and EMM performed the experiments and acquisition of data. KIR, DTB and EMM analyzed the data. All authors participated in writing and revising the manuscript.

## Pre-publication history

The pre-publication history for this paper can be accessed here:

http://www.biomedcentral.com/1471-2342/13/10/prepub
